# Supplementary material for: MetaMap: an atlas of metatranscriptomic reads in human disease-related RNA-seq data
Source: Gigascience. 2018 Jun 12;7(6):giy070. doi: 10.1093/gigascience/giy070 (PMC6025204; doi:10.1093/gigascience/giy070)
Supplement: GIGA-D-18-00063_Revision_1.pdf [file giy070_giga-d-18-00063_revision_1.pdf]

GigaScience

MetaMap: An atlas of metatranscriptomic reads in human disease-related RNA-seq data

--Manuscript Draft--

**Manuscript Number:**	GIGA-D-18-00063R1
**Full Title:**	MetaMap: An atlas of metatranscriptomic reads in human disease-related RNA-seq data
**Article Type:**	Data Note
**Funding Information:**	H2020 Marie Skłodowska-Curie Actions (753039) — Mr. Lukas Simon
**Abstract:**	Background: With the advent of the age of big data in bioinformatics, large volumes of data and high performance computing power enable researchers to perform re-analyses of publicly available datasets at an unprecedented scale. Ever more studies imply the microbiome in both normal human physiology and a wide range of diseases. RNA sequencing technology (RNA-seq) is commonly used to infer global eukaryotic gene expression patterns under defined conditions, including human disease-related contexts, but its generic nature also enables the detection of microbial and viral transcripts.<br>Findings: We developed a bioinformatic pipeline to screen existing human RNA-seq datasets for the presence of microbial and viral reads by re-inspecting the non-human-mapping read fraction. We validated this approach by recapitulating outcomes from 6 independent controlled infection experiments of cell line models and comparison with an alternative metatranscriptomic mapping strategy. We then applied the pipeline to close to 150 terabytes of publicly available raw RNA-seq data from >17,000 samples from >400 studies relevant to human disease using state-of-the-art high performance computing systems. The resulting data of this large-scale re-analysis are made available in the presented MetaMap resource.<br>Conclusions: Our results demonstrate that common human RNA-seq data, including those archived in public repositories, might contain valuable information to correlate microbial and viral detection patterns with diverse diseases. The presented MetaMap database thus provides a rich resource for hypothesis generation towards the role of the microbiome in human disease. Additionally, codes to process new datasets and perform statistical analyses are made available at https://github.com/theislab/MetaMap.
**Corresponding Author:**	Lukas Simon, Ph.D.<br>Helmholtz Zentrum München Deutsches Forschungszentrum für Umwelt und Gesundheit<br>Neuherberg, Bayern GERMANY
**Corresponding Author Secondary Information:**	
**Corresponding Author's Institution:**	Helmholtz Zentrum München Deutsches Forschungszentrum für Umwelt und Gesundheit
**Corresponding Author's Secondary Institution:**	
**First Author:**	Lukas Simon, Ph.D.
**First Author Secondary Information:**	
**Order of Authors:**	Lukas Simon, Ph.D.
	Sebastian Karg
	Alexander Westermann, Dr.
	Marion Engel, Dr.
	Ali Elbehery, Dr.
	Burkhard Hense, Dr.

Powered by Editorial Manager® and ProduXion Manager® from Aries Systems Corporation

	Matthias Heinig, Dr.
	Li Deng, Dr.
	Fabian Theis, Prof. Dr. Dr.
**Order of Authors Secondary Information:**	
**Response to Reviewers:**	Dear Editor,<br><br>We are resubmitting a revised and improved version of our manuscript. We would like to thank you and the reviewers for the valuable feedback. We have considered all points raised by the reviewers and we believe that we have addressed them all.<br>Our point by point answers are the following:<br><br>Reviewer reports:<br>Reviewer #1: This is an interesting idea, implementation and resource, which is useful and valuable. There are some possible confounders in this sort of analysis, they must be spelled out clearly, especially if the resource offers statistical analysis; it is not fully clear from the MS.<br><br>Reply: We appreciate the overall positive comment of the reviewer.<br><br>Changes needed:<br>- State clearly what is offered. Just the datasets of identified non-human reads? Or also the tool to generate equivalent data for new datasets? And further, also the pipeline for statistical analysis?<br>-- At least the second should be offered - scripts and/or tutorial for how to build DB-compatible similar output from a new RNA-seq dataset.<br><br>Reply: We have created a github repository (https://github.com/theislab/MetaMap) which contains a tutorial R script describing the statistical analysis and code to process new data.<br>In total, the manuscript offers three outputs: 1) script to process new datasets with the MetaMap pipeline, 2) the count matrix derived from running the MetaMap pipeline on the described set of >17,000 samples and 3) example code for the statistical analysis.<br>To clarify this, we added the following sentence at the end of the Conclusions section of the abstract: “Additionally, codes to process new datasets and perform statistical analyses are made available at https://github.com/theislab/MetaMap.” In addition, we edited the last sentences of the manuscript as follows: “The composite metafeature OTU count table, derived from 17,278 cDNA libraries from 436 SRA projects, including annotations is provided for download [37]. The MetaMap pipeline and example code for performing statistical analysis can be found at https://github.com/theislab/MetaMap.”<br><br>- If the differential analysis pipeline is not offered to the reader, do not state there is any way to acquire significance for an enrichment of a non-human entity; better yet, provide those scripts.<br>-- If you do however, you must state precisely what significance is being tested, and how that is impacted by the potential issues listed below.<br><br>Reply: We acknowledge the concern of the reviewer regarding technical confounders. This is indeed an important point. As the reviewer pointed out in her last comment, within study case-control comparisons of each metafeature in turn are likely robust with regards to these concerns.<br>Therefore, we have added the following sentence to the Data validation and quality control section:<br>“However, comparisons across studies and metafeatures may be biased by technical confounders (discussed in detail in the Re-use potential section). Therefore, we focussed our analysis on the comparison of a single metafeature across subjects within a study”<br>Additionally, we have added the following detailed discussion of the confounders and recommended analyses to the Re-use potential section:<br>“In addition, technical confounders can obstruct the analysis and potentially generate artificial differences if not considered properly. For example, different types of human samples may contain different amounts of non-human material due to varying sterility

of the tissues. Furthermore, sequencing depth may introduce a detection floor for low abundant metafeatures. Therefore, comparisons across different tissues and sequencing depths may generate artificial differences. Additionally, given that only uniquely discriminative sequences are counted, the absolute abundance levels may not be comparable across metafeatures. Finally, the MetaMap pipeline captures metafeature abundance at the RNA level, which may not necessarily correspond to genomic abundance levels. Metafeatures may be low abundant at the DNA level but highly transcriptionally active and thus abundantly detected at the RNA level, or the inverse. These potential challenges need to be taken into consideration when comparing across metafeatures.
To minimize these effects, we encourage focusing on studies including intra-project comparisons testing each metafeature at a time, such as exemplified in the differential metafeature abundance analysis. Our rationale is that technical confounders - in contrast to biologically meaningful changes - should affect all runs within a project to the same extent and therefore not show condition-specific effects. For example, in the Westermann et al study [33] we detected substantial levels of phiX in both conditions (infected samples and mock-treated controls), but only the 'Salmonella' metafeature showed a condition-specific effect. We aim to address the challenges inherent to inter-project and inter-metafeature comparisons in future work."

- There are potential confounders, and these must be discussed how they impact the sort of analyses you suggest, both generally and in your benchmark.
-- First, different types of human samples will have different amounts of non-human materials because the compartments are more or less sterile, regardless of differential clinical risk etc. This may also reflect purely technical considerations such as risk of contamination for different types of sampling etc. How is this accounted for, and what possible impact may it have?

Reply: The reviewer makes a valid point. Varying compartments can lead to varying sterility. However, we make the assumption that the studies were designed in such a way that technical factors, such as cellular compartment, are not confounded with the conditions being tested. Therefore, we encourage comparisons across samples obtained from the same study which are likely to be robust to these technical confounders. To make this clear we have added detailed discussion to the re-use potential section (see details above).

-- One particularly important effect is that low abundance means a detection floor, where some samples have more things getting above that floor, so one would have a risk of artificial signal resulting just from sample size or technical factors. There would be ways around that but it must be considered and discussed.

Reply: The reviewer makes a valid point. Low abundance can lead to a detection floor. However, we make the assumption that the studies were designed in such a way that technical factors, sequencing depth, are not confounded with the conditions being tested. Therefore, we encourage comparisons across samples obtained from the same study which are likely to be robust to these technical confounders. To make this clear we have added detailed discussion to the re-use potential section (see details above).

-- For RNA being expression data, gene expression (as a transient, condition-dependent thing) will have additional impact beyond the abundance of non-human entitities genomically, however you assess taxonomic composition/presence. This may introduce biases (detected features may be low-abundant but highly active, or the inverse), and different genes in those entities may show up more or less abundantly. This too must be discussed and considered.

Reply: We agree with the reviewer that the manuscript needs to clarify the difference between measuring DNA and RNA abundance. To make this clear we have added detailed discussion to the re-use potential section (see details above).

-- How database-sensitive is the CLARK-S tool? I.e. will many more non-human entities be found if they come from well-studied, well-represented clades? The risk seems larger still if it aims for species-specific marker sequences; it seems then likely to miss entirely sisters or cousins of the already-known features, which would cause artificial difference/contrast.

	Reply: The reviewer makes a good argument. Absolute comparisons across metafeatures can be biased. Therefore, we encourage comparisons of the same metafeature across samples which are likely to be robust to this technical confounder. To make this clear we have added detailed discussion to the re-use potential section (see details above).<br><br>-- How does one normalize these sorts of data for inter-sample comparisons?<br><br>Reply: The data are “normalized” during statistical comparison by including covariates (such as sequencing depth) into the model. We use the DESeq2 R package to perform the statistical comparison and by default DESeq2 normalizes for library size differences by including the estimated size factors as covariates in the model. To clarify this we edited the following sentence in the Data validation and quality control section:<br>“Using the annotation provided in the respective study, we performed differential metafeature abundance analysis to identify those metafeatures that show the largest relative difference in abundance levels between the infected and control samples (see Methods for details).”<br>Moreover, we added the following sentences to the Differential metafeature abundance section: “DESeq2 models differential gene expression by fitting a negative binomial distribution to the raw counts underlying RNA-seq data. This framework can account for confounding variables such as sequencing depth. Therefore, the data need not be normalized prior to statistical inter-sample comparisons.”<br><br>-- For analysing equivalent sample types (e.g. treated/untreated, testing each feature in turn) this is less of a problem, whereas if one e.g. compares viral load in different tissues, or compare prevalence of different viruses, it becomes more of a problem. These are not challenges the authors must solve, necessarily, but to make the tool maximally useful (including as a starting point for future work) these challenges must be discussed and acknowledged in detail.<br><br>Reply: The reviewer has pointed out a number of important challenges that need to be considered during the analysis of this data. We have extended the re-use potential section to discuss the technical confounders and suggest analysis approaches to make the tool maximally useful (see details above).<br><br>Reviewer #2: The authors have developed a valuable database of viral, bacterial and fungal sequences derived from publically available human RNA-seq datasets. They have carefully selected RNA-seq datasets that are not poly-A selected (to avoid datasets that will be primary human based on molecular enrichment). The methodology they present is sound, and the experiments that they designed to validate the data are convincing.<br><br>As noted by the editor the database is not currently available, but once this is amended, the publication looks to be ready. I would also recommend that the protocol in protocols.io is also update with this important information upon publication.<br><br>Reply: We thank the reviewer for the positive review. We have deposited the data set in the GigaScience Database and added the following Availability of supporting data section to the manuscript:<br>“The data sets supporting the results of this article are available in the GigaScience Database repository, http://dx.doi.org/10.5524/100456.”
**Additional Information:**	
**Question**	**Response**
Are you submitting this manuscript to a special series or article collection?	Yes
Please select an option from the menu: as follow-up to "Are you submitting this manuscript to a special series or article collection?"	Functional Metagenomics
**Experimental design and statistics**	Yes

Powered by Editorial Manager® and ProduXion Manager® from Aries Systems Corporation

Full details of the experimental design and statistical methods used should be given in the Methods section, as detailed in our Minimum Standards Reporting Checklist. Information essential to interpreting the data presented should be made available in the figure legends.<br><br>Have you included all the information requested in your manuscript?	
**Resources**<br><br>A description of all resources used, including antibodies, cell lines, animals and software tools, with enough information to allow them to be uniquely identified, should be included in the Methods section. Authors are strongly encouraged to cite Research Resource Identifiers (RRIDs) for antibodies, model organisms and tools, where possible.<br><br>Have you included the information requested as detailed in our Minimum Standards Reporting Checklist?	Yes
**Availability of data and materials**<br><br>All datasets and code on which the conclusions of the paper rely must be either included in your submission or deposited in publicly available repositories (where available and ethically appropriate), referencing such data using a unique identifier in the references and in the "Availability of Data and Materials" section of your manuscript.<br><br>Have you have met the above requirement as detailed in our Minimum Standards Reporting Checklist?	Yes

Manuscript

Click here to download Manuscript
Manuscript_revised_v2.docx

Title

MetaMap: An atlas of metatranscriptomic reads in human disease-related RNA-seq data

Authors

Simon LM1, Karg S1, Westermann AJ2,3, Engel M1,4, Elbehery AHA5, Hense B1, Heinig M1, Deng L5, Theis FJ1,6

Affiliations

1 Helmholtz Zentrum München, German Research Center for Environmental Health, Institute of Computational Biology, Neuherberg, Germany

2 Institute of Molecular Infection Biology (IMIB), University of Würzburg, Würzburg, Germany

3 Helmholtz Institute for RNA-Based Infection Research (HIRI), Würzburg, Germany

4 Helmholtz Zentrum München, German Research Center for Environmental Health, Scientific Computing Research Unit, Neuherberg, Germany

5 Helmholtz Zentrum München, German Research Center for Environmental Health, Institute of Virology, Neuherberg, Germany

6 Department of Mathematics, Technische Universität München, Munich, Germany

Corresponding authors

Simon LM; lukas.simon@helmholtz-muenchen.de, ORCID: 0000-0001-6148-8861

Theis FJ; fabian.theis@helmholtz-muenchen.de, ORCID: 0000-0002-2419-1943

Abstract

Background: With the advent of the age of big data in bioinformatics, large volumes of data and high performance computing power enable researchers to perform re-analyses of publicly available datasets at an unprecedented scale. Ever more studies imply the microbiome in both normal human physiology and a wide range of diseases. RNA sequencing technology (RNA-seq) is commonly used to infer global eukaryotic gene expression patterns under defined conditions, including human disease-related contexts, but its generic nature also enables the detection of microbial and viral transcripts.

Findings: We developed a bioinformatic pipeline to screen existing human RNA-seq datasets for the presence of microbial and viral reads by re-inspecting the non-human-mapping read fraction. We validated this approach by recapitulating outcomes from 6 independent controlled infection experiments of cell line models and comparison with an alternative metatranscriptomic mapping strategy. We then applied the pipeline to close to 150 terabytes of publicly available raw RNA-seq data from >17,000 samples from >400 studies relevant to human disease using state-of-the-art high performance computing systems. The resulting data of this large-scale re-analysis are made available in the presented MetaMap resource.

Conclusions: Our results demonstrate that common human RNA-seq data, including those archived in public repositories, might contain valuable information to correlate microbial and viral detection patterns with diverse diseases. The presented MetaMap database thus provides a rich resource for hypothesis generation towards the role of the microbiome in human disease. Additionally, codes to process new datasets and perform statistical analyses are made available at https://github.com/theislab/MetaMap.

Keywords

High performance computing, big data, RNA-seq, sequence read archive, metatranscriptomics, microbiome, virome, human disease, infection

Data Description

Context

Recent studies have demonstrated the paramount importance of the microbiome for human health and disease [1]. For example, imbalance of the human gut microbiome was linked to non-communicable diseases such as obesity [2,3], diabetes [4], cardiovascular disease [5], chronic obstructive pulmonary disease [6], or colorectal carcinoma [7,8], to name just a few.

The advent of high-throughput sequencing technologies has revolutionized the life sciences. RNA-seq technology produces one of the most frequent next generation sequencing data types and has been applied to study a large number of biological samples relevant to human disease. The majority of the underlying raw data is freely accessible from data repositories such as the Gene Expression Omnibus (GEO) (>1,700 human RNA-seq data sets as of january 2018) or the Sequence Read Archive (SRA) [9].

However, these data are typically exclusively used for single species (i.e. human) transcriptomics such as differential gene expression or alternative splicing analysis [9,10]. Reads that do not map onto the human genome are considered noise or contamination and therefore generally ignored [11,12] (collectively about 9% of total reads, Fig. 1). Five years ago, it was postulated that interspecies interactions might be studied by simultaneous detection and quantification of RNA transcripts from a given host and a microbe via ‘dual’ RNA-seq [13]. Meanwhile this approach has been successfully applied to the interaction of mammalian cells with diverse bacterial [14] and viral pathogens [15–19].

Inspired by dual RNA-seq, in this study we hypothesize that reads in archived RNA-seq datasets derived from human primary cells or tissue samples that fail to map against the human reference genome may contain valuable information about the presence of certain microbes in the respective body niches and/or under defined disease conditions. To enable metatranscriptomic study of these data, we combined existing read alignment and metagenomic classification software into a two-step ‘omni’ RNA-seq pipeline to

comprehensively quantify archaeal, bacterial and viral reads in human RNA-seq data (Fig. 1).

In the first step of this so called ‘Metamap’ pipeline, all reads are aligned against the human genome using the ultra-fast RNA-seq aligner STAR [20] and subsequently only the fraction of unmapped reads is subjected to metatranscriptomic classification using CLARK-S [21] (see Methods for details). The combination between scalability and accuracy was the main motivation behind choosing these two software packages over competing methods [22,23]. It is important to note that CLARK-S uses a set of uniquely discriminative short sequences at the species level to classify reads. Therefore, reads containing non-discriminative sequences that fail to be uniquely assigned to a single species, e.g. reads originating from the bacterial ribosomal 16S rRNA gene, will be considered ‘unclassified’ (altogether 8.6% in Fig. 1).

The output of CLARK-S is an operational taxonomic units (OTU) count matrix, where rows correspond to viral, bacterial and archeal species and columns to (human) samples. Each entry corresponds to the number of non-human reads classified to the respective species. For convenience, in the following we refer to the set of microbial and viral species profiled using our approach as ‘metafeatures’.

By screening the study abstracts of the SRA for search terms prioritizing human clinical datasets derived from polyA-independent sequencing protocols (see Methods) we identified over 400 studies relevant to human disease comprising more than 17,000 cDNA libraries (close to 150 terabytes of raw sequencing data). Raw sequencing reads from these studies were downloaded and analyzed using the high performance computing system of the Leibniz Supercomputing Centre (LRZ) of the Bavarian Academy of Sciences and Humanities which facilitated ultra-fast processing with median speeds of 25 and 21 million reads per hour per core per run for the STAR and CLARK-S steps, respectively. Overall, of the total over 500 billion RNA-seq reads processed, around 91% could be mapped to the human genome. A fraction of 8.6% of all reads remained non-discriminative at the species level and is defined as “unclassified”. 0.03%, 0.20% and 0.39% of all reads were assigned to archaeal, bacterial

or viral metafeatures, respectively. Despite these relatively low percentages, the absolute numbers of reads classified were in the hundred millions to billions, enabling statistical analyses.

Methods

High performance computing environment. Project computations including download, alignment of reads onto the human genome and metafeature quantification were made on the high performance Linux Cluster at the LRZ (www.lrz.de/services/compute/linux-cluster).

RNA-seq data retrieval. Raw next generation sequencing data were downloaded from the SRA. The R package *SRAdb* was downloaded on 23 May 2017 and used to query of the SRA database. To identify SRA projects that contain transcriptomic analyses of human RNA-seq data, the SRA attributes 'taxon_id', 'library_source', 'library_strategy', 'platform' were searched for the terms '9606', 'TRANSCRIPT', 'RNA-seq', 'ILLUMINA', respectively. To remove potential bias derived from different sequencing technologies we also restricted the query to SRA runs annotated with 'ILLUMINA' in SRA attribute 'platform'. To exclude studies with insufficient sample size for statistical analysis the query was restricted to SRA projects containing more than five runs. To avoid concentrating the analysis on a small number of large projects the query was restricted to SRA projects with less than 500 runs. To identify studies focusing on phenotypes relevant to human disease, we restricted the query to runs containing at least one or more of the terms 'disease', 'patient', 'primary' and 'clinical' in the SRA attribute 'study_abstract'. To exclude *in vitro* (cell-culture) experiments, but focus on primary (clinical) samples, SRA runs containing the terms "mutant" or "cell-line" were removed from our selection. Furthermore, SRA runs containing the terms "single cell" and "GTEx" were removed. Finally, samples with less than 1 million total reads or read lengths <50 base pairs were excluded. The described query resulted in 484 Short Read Projects (SRPs) containing a total of 21,659 RNA-seq runs. Due to technical problems (i.e. missing URLs, restricted access) we were unable to download a fraction of 4,078 samples.

Human alignment. Alignment of reads against the human reference genome (hg38) and simultaneous human gene expression quantification was conducted with STAR (version 2.5.2). To increase mapping speed of a large number of samples, we used the --*genomeLoad LoadAndKeep* function to load the STAR index once and keep it in memory for subsequent alignments. The parameter --*quantmode GeneCounts* was used to generate the human gene expression count tables. Unmapped reads were saved with the --*outReadsUnmapped Fastx* parameter. To further increase mapping speed, multiple threads were used as implemented with the parameter *--runThreadN 28*. Runs with less than 30% reads mapping to the human genome were excluded from downstream analysis. All human alignments were conducted on the LRZ "CoolMUC2" Linux-Cluster. This cluster contains 384 nodes with 64 GB RAM memory and 28 cores each.

Metafeature quantification. Metafeature quantification was conducted with CLARK-S (version 1.2.3). CLARK-S is a software method for fast and accurate sequence classification of metagenomic next-generation sequencing data, including RNA-seq data. One major issue during the classification of metagenomic data is the rising number of targets to align against. CLARK-S solves this issue by building a large index file consisting of discriminative *k*-mers. The metagenomic reference database was generated following the description of the CLARK website using the following two commands: 1) *set_targets.sh bacteria virus --species* and 2) *buildSpacedDB.sh*. This database contained a total of 16,551 genome sequences corresponding to 6,979 unique species (additional file 2). To allow uniform processing, paired-end sequencing experiments were analyzed independently. Each single unmapped reads file was used as input for CLARK-S with the following parameters: *classify_metagenome.sh --spaced –O* list of FASTQ files. To increase classification speed, the CLARK-S express mode was selected and multiple threads were used with parameters --*m 2* and --*n 32*, respectively. The output files of this step contain all input read identifiers with the corresponding metafeature classification. In the subsequent step, total counts are summarized for each feature with the *estimate_abundance.sh* command. To enable comparison across single-end and paired-end experiments, metafeature counts from paired-

end experiments were averaged and subsequently rounded to conserve count distribution. To account for varying sequencing depths, metafeature abundance was estimated as the number of reads per million (RPM) total reads sequenced. Metafeature quantification was conducted on the LRZ “Teramem” Linux-Cluster. This cluster contains one node with 6,144 GB RAM memory and 96 cores.

BLAST based metafeature classification. To validate results generated by the MetaMap pipeline, the Basic Local Alignment Search Tool [24] was used as follows. A BLAST database was created from the same genome sequences used in the CLARK-S approach. Then, reads were aligned to this database using BLASTN with a threshold E-value of 1e-10. Produced counts from paired-end experiments were averaged. For each file, BLAST was done by running approximately 10 kilobase chunks (record separator ">") in parallel using GNU parallel (28 jobs), each with 8 threads using one node on the LRZ “CoolMUC3” Linux Cluster. This cluster contains 148 nodes with 96 GB RAM memory and 64 cores each. Output was parsed to exclusively keep reads that could be assigned at the species level.

Differential metafeature abundance. Differential metafeature abundance analysis was performed using the R package DESeq2 [25]. DESeq2 models differential gene expression by fitting a negative binomial distribution to the raw counts underlying RNA-seq data. This framework can account for confounding variables such as sequencing depth. Therefore, the data need not be normalized prior to statistical inter-sample comparisons. For each of the four published bona fide dual RNA-seq studies we classified samples into two groups based on the provided annotations: 1) Samples expected to contain the known pathogen, such as human papillomavirus positive tumors in the Zhang et al study, and 2) pathogen-free controls, such as mock-treated cells in the Westermann et al study. Using this binary outcome we performed differential expression analysis across all detected metafeatures. To account for sequencing depth, library size factors were estimated from the total number of sequenced reads. The dispersion for the negative binomial distribution was estimated using a local linear regression as implemented in the *DESeq()* function via the *fitType* parameter ‘local’.

Data validation and quality control

We validated our approach by recovering the ground truth in bona fide dual RNA-seq experiments performed with human cell lines and samples from patients with well-known infection status. Of the four selected studies, one analyzed an infection model based on a bacterial (*Salmonella enterica* serovar Typhimurium) and three based on distinct viral pathogens (Human papillomavirus, Herpes simplex virus, Rhinovirus). As expected, MetaMap detected the known pathogen at higher levels in the respective study compared to the other studies and pathogens (Table 1). However, comparisons across studies and metafeatures may be biased by technical confounders (discussed in detail in the Re-use potential section). Therefore, we focussed our analysis on the comparison of a single metafeature across subjects within a study. Using the annotation provided in the respective study, we performed differential metafeature abundance analysis to identify those metafeatures that show the largest relative difference in abundance levels between the infected and control samples (see Methods for details). The correct infection agent showed the most significant difference across all metafeatures between infected and control samples for each study (Fig. 2). For example, Westermann et al [26] generated dual RNA-seq data from HeLa cells infected with the enteric bacterial pathogen *Salmonella enterica* serovar Typhimurium and compared them to mock-treated control samples. Accordingly, we here observed *Salmonella enterica* as the most differentially abundant metafeature between the infected and the control samples ($P<1e-75$, Fig. 2A). Likewise we recovered *Alphapapillomavirus 9*, *Human alphaherpesvirus 1* (also known as herpes simplex virus 1) and *Rhinovirus A* as the most differentially abundant metafeatures in the data from Zhang et al [27], Rutkowski et al [28] and Bai et al [29], respectively. In the Westermann et al [26] and Rutkowski et al [28] studies, several additional metafeatures showed a strong differential abundance effect (Fig. 2A & C). These metafeatures were closely related to the true infection agent, i.e *Salmonella bongori* ($P<1e-67$) and *Panine alphaherpesvirus 3* ($P<1e-9$) for the Westermann et al [26] or Rutkowski et al [28] study, respectively. These findings confirm that

our MetaMap pipeline recapitulates results from dedicated dual RNA-seq studies, i.e. studies based on known infectious agents. Therefore, MetaMap may be equally suited to detect previously unknown microbial and viral species in human primary samples.

As an additional control, we re-analysed two projects contained in our data collection that are derived from the B lymphoblast cell line, under non-infectious conditions. However, since Epstein-Barr virus is used for transfection and transformation of lymphocytes to lymphoblasts, we expected to detect reads from this virus in these projects [30], but no further viral or microbial reads [31]. Indeed the most abundant metafeatures in each project were dominated by reads classified to *Gammaherpesvirus 4* (also known as Epstein-Barr virus, EBV) and *Enterobacteria phage phiX174 sensu lato* (phiX), commonly used as spike-in in Illumina sequencing runs [32] (Fig. 3A-B). On average 95% and 97% of all metafeature reads were classified as phiX or EBV for projects SRP041338 and SRP091453, respectively (Fig. 3C). Conversely, the abundance of reads mapping to bacterial species for these two projects corresponds to the bottom percentile as compared to all other projects in the MetaMap database, supporting sterility of this cell line (Fig. 3D). This demonstrates that MetaMap not only is capable of re-discovering known pathogenic species (true positives) in controlled infection experiments (Fig. 2), but it also minimizes the detection of false positives or at least, provides measures such as abundance and significance allowing the user to identify and counterselect those species.

As a technical validation, we compared our approach to an alternative metatranscriptomic classification strategy for the Westermann et al [33] study. All non-human reads were aligned using BLASTN to a BLAST database consisting of the same genomic sequences used by CLARK-S (see Methods for details). The average metafeature abundances across all 42 samples derived from the BLAST based approach and CLARK-S correlated significantly (Spearman correlation, Rho: 0.16, P: 3.1e-10) (Fig. 4A). BLAST showed higher sensitivity and detected more metafeatures compared to CLARK-S (indicated by the accumulation of dots at value 0 on the X-axis in Fig. 4A). This is mostly observed for low abundance metafeatures which could represent low counts derived from sequencing

and/or mapping errors. However, most importantly the true pathogen metafeature '*Salmonella enterica*' showed very high correlation across samples between the BLAST and CLARK-based abundance estimates (Fig. 4B). Noteworthy, the MetaMap pipeline processed reads more than three orders of magnitude faster than BLAST, demonstrating a significant speed advantage while generating comparable results (Fig. 4C).

Re-use potential

Microbial and viral contamination in next-generation sequencing data was observed before. It can be caused by mapping errors due to genome sequence similarity between different species [34,35]. In addition, technical confounders can obstruct the analysis and potentially generate artificial differences if not considered properly. For example, different types of human samples may contain different amounts of non-human material due to varying sterility of the tissues. Furthermore, sequencing depth may introduce a detection floor for low abundant metafeatures. Therefore, comparisons across different tissues and sequencing depths may generate artificial differences. Additionally, given that only uniquely discriminative sequences are counted, the absolute abundance levels may not be comparable across metafeatures. Finally, the MetaMap pipeline captures metafeature abundance at the RNA level, which may not necessarily correspond to genomic abundance levels. Metafeatures may be low abundant at the DNA level but highly transcriptionally active and thus abundantly detected at the RNA level, or the inverse. These potential challenges need to be taken into consideration when comparing across metafeatures.

To minimize these effects, we encourage focusing on studies including intra-project comparisons testing each metafeature at a time, such as exemplified in the differential metafeature abundance analysis. Our rationale is that technical confounders - in contrast to biologically meaningful changes - should affect all runs within a project to the same extent and therefore not show condition-specific effects. For example, in the Westermann et al study [33] we detected substantial levels of phiX in both conditions (infected samples and mock-treated controls), but only the '*Salmonella*' metafeature showed a condition-specific

effect. We aim to address the challenges inherent to inter-project and inter-metafeature comparisons in future work.

All the raw data described in the present study were publicly available before, yet have been very cumbersome to extract individually. The presented MetaMap database now makes these data easily accessible for a very broad community, thereby allowing for global comparisons over hundreds of individual studies and thousands of sampled conditions. While we attempted to minimize the risk of detecting false positives (Fig. 3), it should be noted that not all metafeatures classified by MetaMap will necessarily refer to true biological factors. Noteworthy, our approach reveals correlation between metafeatures and disease, not causality, and cannot discriminate disease-associated effects from potential treatment effects. However, our pipeline provides the user with a scientific starting ground to validate the presence/absence of defined microbial and viral species under defined conditions and explore the underlying biology and significance in greater detail. As a potential use case of these data, users can test for associations of microbial or viral metafeatures with a plethora of human diseases, or between themselves. In addition, users with interest in a specific bacterial or viral species can easily identify studies, and consequently disease contexts, in which reads from this organism were detected. This could give an important first hint to assess whether the respective species might be implicated in a given human disease etiology. Furthermore, this resource provides the opportunity to support findings derived from standard microbiome profiling technologies, such as 16S rRNA gene based or shotgun metagenomics [36]. Finally, metafeature detection in human clinical RNA-seq samples may provide a diagnostic advantage when studying microbes or viruses which are challenging to isolate.

The composite metafeature OTU count table, derived from 17,278 cDNA libraries from 436 SRA projects, including annotations is provided for download [37]. The MetaMap pipeline and example code for performing statistical analysis can be found at https://github.com/theislab/MetaMap.

Figure legends

Figure 1. Schematic illustrates the MetaMap pipeline. Over 400 projects from studies relevant to human disease were identified in the SRA database. Over 500 billion RNA-seq reads were downloaded and first filtered by mapping them onto the human genome and subsequently the remaining reads underwent metafeature classification. 90.7% of all reads mapped to the human genome. 0.03%, 0.20% and 0.39% of all reads were assigned to archaeal, bacterial or viral metafeatures, respectively. 8.6% of all reads remain non-discriminative at the species level ('unclassified').

Figure 2. Differential metafeature abundance analysis of controlled infection experiments recovers ground truth. Panels A-D depict "volcano" plots showing fold change and inverted p-value on the X and Y axes, respectively. Each dot represents a metafeature. The most significant metafeature is colored in red. Insets display boxplots of the abundance levels in RPM of the top hit metafeature across conditions for each study. For all boxplots, the box represents the interquartile range, the horizontal line in the box is the median, and the whiskers represent 1.5 times the interquartile range.

Figure 3. Analysis of lymphoblast cell line experiments further supports the MetaMap pipeline. Panels A and B depict mean abundance levels across all samples of the top five metafeatures for projects SRP041338 and SRP091453, respectively. Panel C shows relative proportion of reads mapping to EBV, phiX and all other metafeatures across RNA-seq samples. Panel D depicts the cumulative distribution plot of the average proportion of bacterial metafeature reads across all projects. Purple and pink vertical lines highlight projects SRP041338 and SRP091453, respectively.

Figure 4. Alternative BLAST-based classification method validates metafeature abundance estimates by MetaMap. Panel A depicts average metafeature RPM levels derived using the CLARK-S software, as implemented in the MetaMap pipeline, and a BLAST-based alternative approach on the X- and Y-axes, respectively. Panel B shows the correlation in *Salmonella enterica* abundance levels between the two classification approaches. Panel C shows the difference in classification speed between the BLAST and CLARK-S metatranscriptomic classification. Y axis shows the number of reads processed per hour per thread in log10 space.

Tables

Study	Infection agent	Total reads	***Salmonella enterica***	**Alphapapillomavirus 9**	**H. alphaherpesvirus 1**	**Rhinovirus A**
Westermann et al	*Salmonella enterica* serovar Typhimurium	1.0e+07	**6.3e+03**	1.2e-01	1.5e-01	1.2e-01
Zhang et al	Human papillomavirus	4.6e+07	3.0e-02	**5.1e+01**	2.2e-02	2.2e-02
Rutkowski et al	Herpes simplex virus	3.5e+07	1.1e+00	3.1e-02	**3.1e+04**	3.0e-02
Bai et al	Rhinovirus	6.6e+06	2.0e-01	1.5e-01	1.5e-01	**4.4e+01**

Table 1. Overview of four dual RNA-seq studies used to validate the MetaMap pipeline. Total reads column depicts the average read depth per sample for each study. Average metafeature abundance for *Alphapapillomavirus 9*, *Salmonella enterica*, *Human alphaherpesvirus 1* and *Rhinovirus A* are shown in RPM. The correct infection agent for the respective study is highlighted in bold font.

Availability of source code and requirements

Project name: MetaMap

Project home: https://github.com/theislab/MetaMap

Operating system(s): Platform-independent

Programming language: Unix command line, *R*

Other requirements: STAR and CLARK-S may require large amounts of memory (>100 GB)

License: GNU GPL

Availability of supporting data

The data sets supporting the results of this article are available in the GigaScience Database repository [37]. The protocols are also available from protocols.io [38].

List of abbreviations

SRA: Sequencing read archive; LRZ: Leibniz Rechenzentrum; GEO: Gene expression omnibus, EBV: Epstein-Barr virus; BLAST: Basic local alignment search tool; STAR: Spliced Transcripts Alignment to a Reference software; OTU: operational taxonomic units; phiX: *Enterobacteria* phage phiX174 sensu lato; RPM: reads per million total reads

Competing interests

Authors declare that they have no competing interests.

Funding

LS acknowledges funding from the European Union's Horizon 2020 research and innovation programme under the Marie Sklodowska-Curie grant agreement No 753039. The operation of the LRZ linux cluster is funded via the Bavarian State Ministry of Education, Science and the Arts.

Author's contributions

Conceptualization, L.S., M.E., L.D., B.H.; Formal analysis, L.S., S.K., A.E.; Investigation, L.S., A.J.W., M.E.; Methodology; Writing - original draft, L.S., A.J.W.; Writing - review & editing, L.S., A.J.W., M.E., A.E., L.D., M.H., F.T.; Supervision, L.D., M.H., F.T.

Acknowledgments

The authors would like to thank Yu Wang and Ferdinand Jamitzky from the LRZ for their support.

References

1. Young VB. The role of the microbiome in human health and disease: an introduction for clinicians. BMJ. 2017;356: j831.

2. Turnbaugh PJ, Ley RE, Mahowald MA, Magrini V, Mardis ER, Gordon JI. An obesity-associated gut microbiome with increased capacity for energy harvest. Nature. 2006;444: 1027–1031.

3. Henao-Mejia J, Elinav E, Jin C, Hao L, Mehal WZ, Strowig T, et al. Inflammasome-mediated dysbiosis regulates progression of NAFLD and obesity. Nature. 2012;482: 179–185.

4. Cani PD, Bibiloni R, Knauf C, Waget A, Neyrinck AM, Delzenne NM, et al. Changes in gut microbiota control metabolic endotoxemia-induced inflammation in high-fat diet-induced obesity and diabetes in mice. Diabetes. 2008;57: 1470–1481.

5. Wang Z, Klipfell E, Bennett BJ, Koeth R, Levison BS, Dugar B, et al. Gut flora metabolism of phosphatidylcholine promotes cardiovascular disease. Nature. 2011;472: 57–63.

6. Engel M, Endesfelder D, Schloter-Hai B, Kublik S, Granitsiotis MS, Boschetto P, et al. Influence of lung CT changes in chronic obstructive pulmonary disease (COPD) on the human lung microbiome. PLoS One. 2017;12: e0180859.

7. Kostic AD, Gevers D, Pedamallu CS, Michaud M, Duke F, Earl AM, et al. Genomic analysis identifies association of Fusobacterium with colorectal carcinoma. Genome Res. 2012;22: 292–298.

8. Castellarin M, Warren RL, Freeman JD, Dreolini L, Krzywinski M, Strauss J, et al. Fusobacterium nucleatum infection is prevalent in human colorectal carcinoma. Genome Res. 2012;22: 299–306.

9. Kodama Y, Shumway M, Leinonen R, International Nucleotide Sequence Database Collaboration. The Sequence Read Archive: explosive growth of sequencing data. Nucleic Acids Res. 2012;40: D54–6.

10. Conesa A, Madrigal P, Tarazona S, Gomez-Cabrero D, Cervera A, McPherson A, et al. A survey of best practices for RNA-seq data analysis. Genome Biol. 2016;17: 13.

11. Gouin A, Legeai F, Nouhaud P, Whibley A, Simon J-C, Lemaitre C. Whole-genome re-sequencing of non-model organisms: lessons from unmapped reads. Heredity . 2015;114: 494–501.

12. Peng X, Wang J, Zhang Z, Xiao Q, Li M, Pan Y. Re-alignment of the unmapped reads with base quality score. BMC Bioinformatics. 2015;16 Suppl 5: S8.

13. Westermann AJ, Gorski SA, Vogel J. Dual RNA-seq of pathogen and host. Nat Rev Microbiol. 2012;10: 618–630.

14. Westermann AJ, Barquist L, Vogel J. Resolving host-pathogen interactions by dual RNA-seq. PLoS Pathog. 2017;13: e1006033.

15. Juranic Lisnic V, Babic Cac M, Lisnic B, Trsan T, Mefferd A, Das Mukhopadhyay C, et al. Dual analysis of the murine cytomegalovirus and host cell transcriptomes reveal new aspects of the virus-host cell interface. PLoS Pathog. 2013;9: e1003611.

16. Xu G, Strong MJ, Lacey MR, Baribault C, Flemington EK, Taylor CM. RNA CoMPASS: a dual approach for pathogen and host transcriptome analysis of RNA-seq datasets. PLoS One. 2014;9: e89445.

17. Park S-J, Kumar M, Kwon H-I, Seong R-K, Han K, Song J-M, et al. Dynamic changes in host gene expression associated with H5N8 avian influenza virus infection in mice. Sci Rep. 2015;5: 16512.

18. Saxena K, Simon LM, Zeng X-L, Blutt SE, Crawford SE, Sastri NP, et al. A paradox of transcriptional and functional innate interferon responses of human intestinal enteroids to enteric virus infection. Proceedings of the National Academy of Sciences. 2017;114: E570–E579.

19. Wesolowska-Andersen A, Everman JL, Davidson R, Rios C, Herrin R, Eng C, et al. Dual RNA-seq reveals viral infections in asthmatic children without respiratory illness which are associated with changes in the airway transcriptome. Genome Biol. 2017;18: 12.

20. Dobin A, Davis CA, Schlesinger F, Drenkow J, Zaleski C, Jha S, et al. STAR: ultrafast universal RNA-seq aligner. Bioinformatics. 2012;29: 15–21.

21. Ounit R, Lonardi S. Higher classification sensitivity of short metagenomic reads with CLARK-S. Bioinformatics. 2016;32: 3823–3825.

22. Lindgreen S, Adair KL, Gardner PP. An evaluation of the accuracy and speed of metagenome analysis tools. Sci Rep. 2016;6: 19233.

23. Engström PG, Steijger T, Sipos B, Grant GR, Kahles A, Rätsch G, et al. Systematic evaluation of spliced alignment programs for RNA-seq data. Nat Methods. 2013;10: 1185–1191.

24. Altschul S. Basic Local Alignment Search Tool. J Mol Biol. 1990;215: 403–410.

25. Love MI, Huber W, Anders S. Moderated estimation of fold change and dispersion for RNA-seq data with DESeq2 [Internet]. 2014. doi:10.1101/002832

26. Westermann AJ, Förstner KU, Amman F, Barquist L, Chao Y, Schulte LN, et al. Dual RNA-seq unveils noncoding RNA functions in host–pathogen interactions. Nature. 2016;529: 496–501.

27. Zhang Y, Koneva LA, Virani S, Arthur AE, Virani A, Hall PB, et al. Subtypes of HPV-Positive Head and Neck Cancers Are Associated with HPV Characteristics, Copy Number Alterations, PIK3CA Mutation, and Pathway Signatures. Clin Cancer Res. 2016;22: 4735–4745.

28. Rutkowski AJ, Erhard F, L'Hernault A, Bonfert T, Schilhabel M, Crump C, et al. Widespread disruption of host transcription termination in HSV-1 infection. Nat Commun. 2015;6: 7126.

29. Bai J, Smock SL, Jackson GR Jr, MacIsaac KD, Huang Y, Mankus C, et al. Phenotypic responses of differentiated asthmatic human airway epithelial cultures to rhinovirus. PLoS One. 2015;10: e0118286.

30. Santpere G, Darre F, Blanco S, Alcami A, Villoslada P, Mar Albà M, et al. Genome-Wide Analysis of Wild-Type Epstein–Barr Virus Genomes Derived from Healthy Individuals of the 1000 Genomes Project. Genome Biol Evol. 2014;6: 846–860.

31. Mangul S, Olde Loohuis LM, Ori A, Jospin G, Koslicki D, Yang HT, et al. Total RNA Sequencing reveals microbial communities in human blood and disease specific effects [Internet]. 2016. doi:10.1101/057570

32. Mukherjee S, Huntemann M, Ivanova N, Kyrpides NC, Pati A. Large-scale contamination of microbial isolate genomes by Illumina PhiX control. Stand Genomic Sci. 2015;10: 18.

33. Westermann AJ, Förstner KU, Amman F, Barquist L, Chao Y, Schulte LN, et al. Dual RNA-seq unveils noncoding RNA functions in host-pathogen interactions. Nature. 2016;529: 496–501.

34. Strong MJ, Xu G, Morici L, Splinter Bon-Durant S, Baddoo M, Lin Z, et al. Microbial contamination in next generation sequencing: implications for sequence-based analysis of clinical samples. PLoS Pathog. 2014;10: e1004437.

35. Bonfert T, Csaba G, Zimmer R, Friedel CC. Mining RNA–Seq Data for Infections and Contaminations. PLoS One. 2013;8: e73071.

36. Cox MJ, W O C, Moffatt MF. Sequencing the human microbiome in health and disease. Hum Mol Genet. 2013;22: R88–R94.

37. Simon, L, M; Karg, S; Westermann, A; Engel, M; Elbehery, A; Hense, B; Heinig, M; Deng, L; Theis, F(2018): Supporting data for "MetaMap: An atlas of metatranscriptomic reads in human disease-related RNA-seq data" GigaScience Database. http://dx.doi.org/10.5524/100456

38. Simon LM, Karg S. MetaMap pipeline. protocols.io. 2018; doi: dx.doi.org/10.17504/protocols.io.msec6be

Figure1

Click here to download Figure Figure1.eps

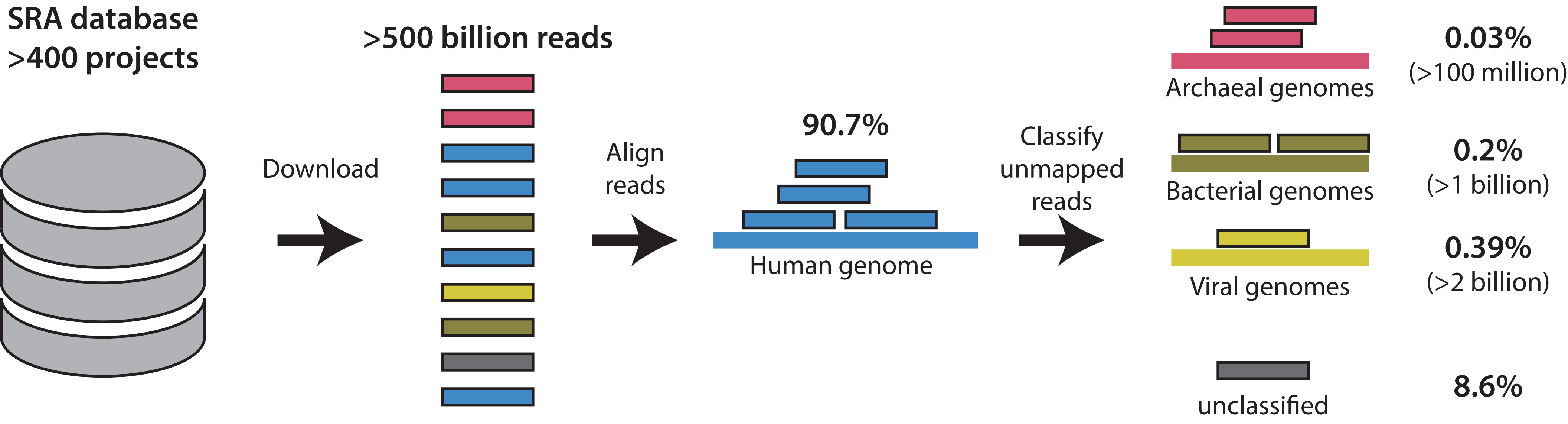

Figure2

Click here to download Figure Figure2.eps

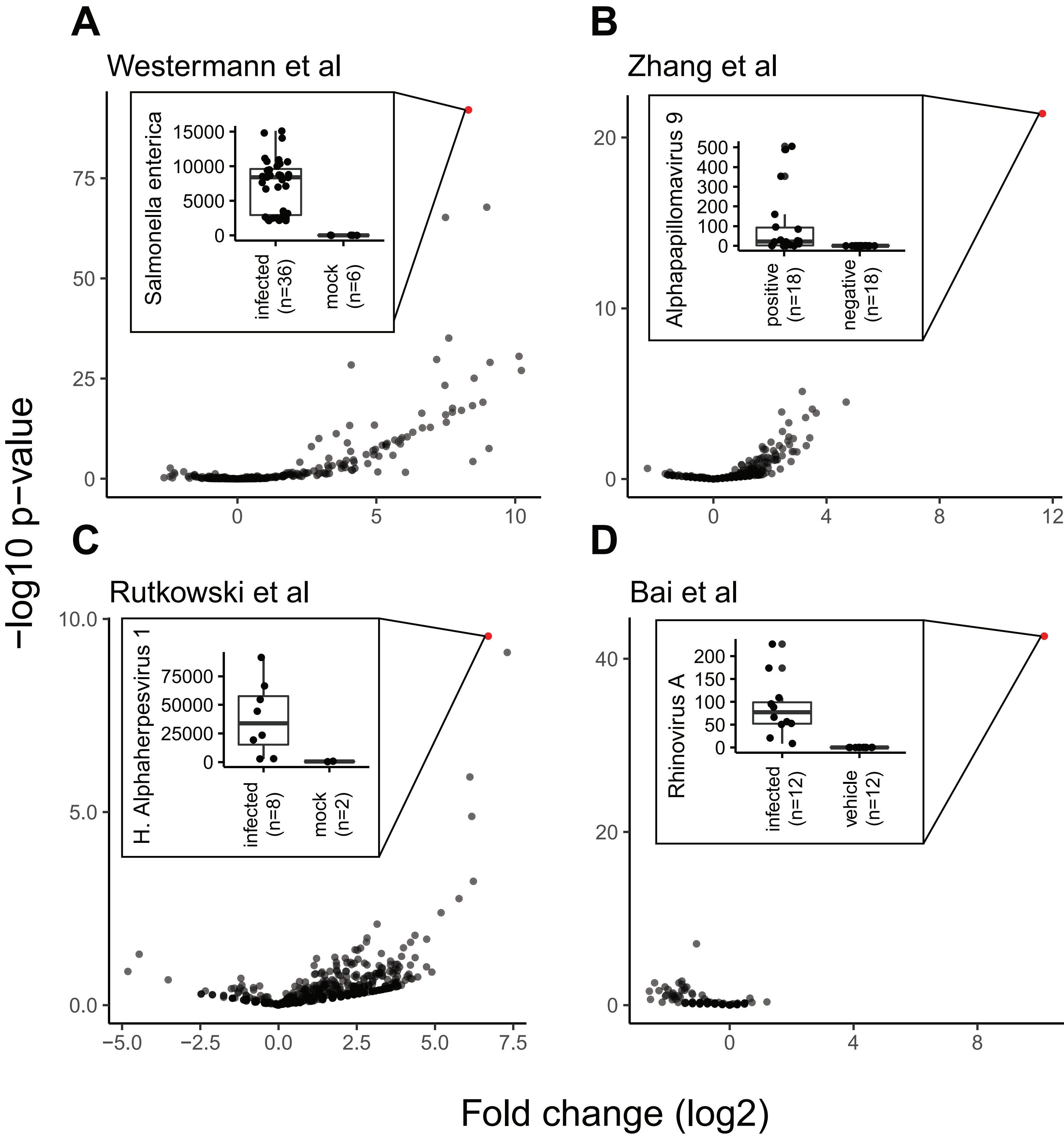

Figure3

Click here to download Figure Figure3.eps

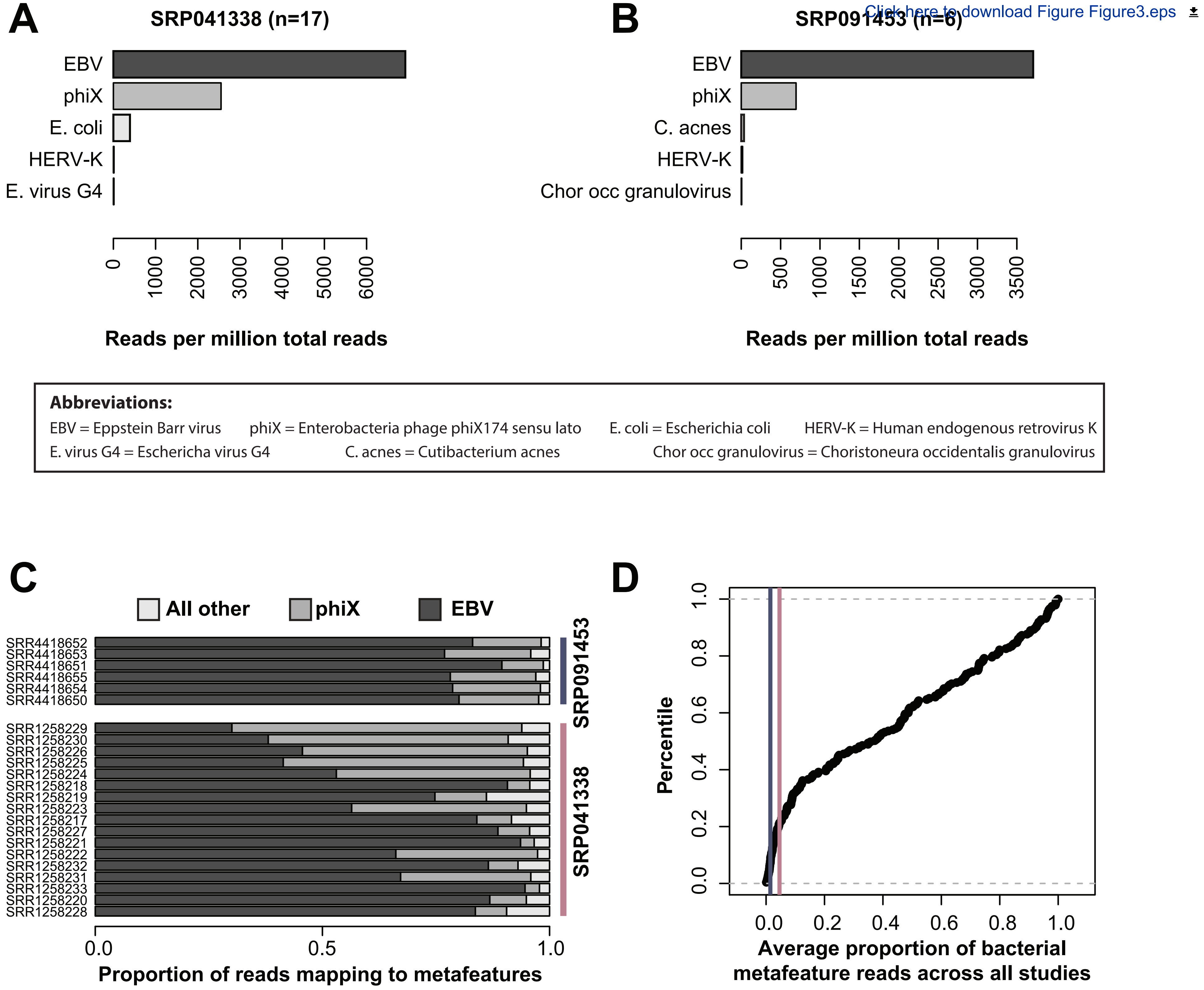

Figure4

Click here to download Figure Figure4.eps

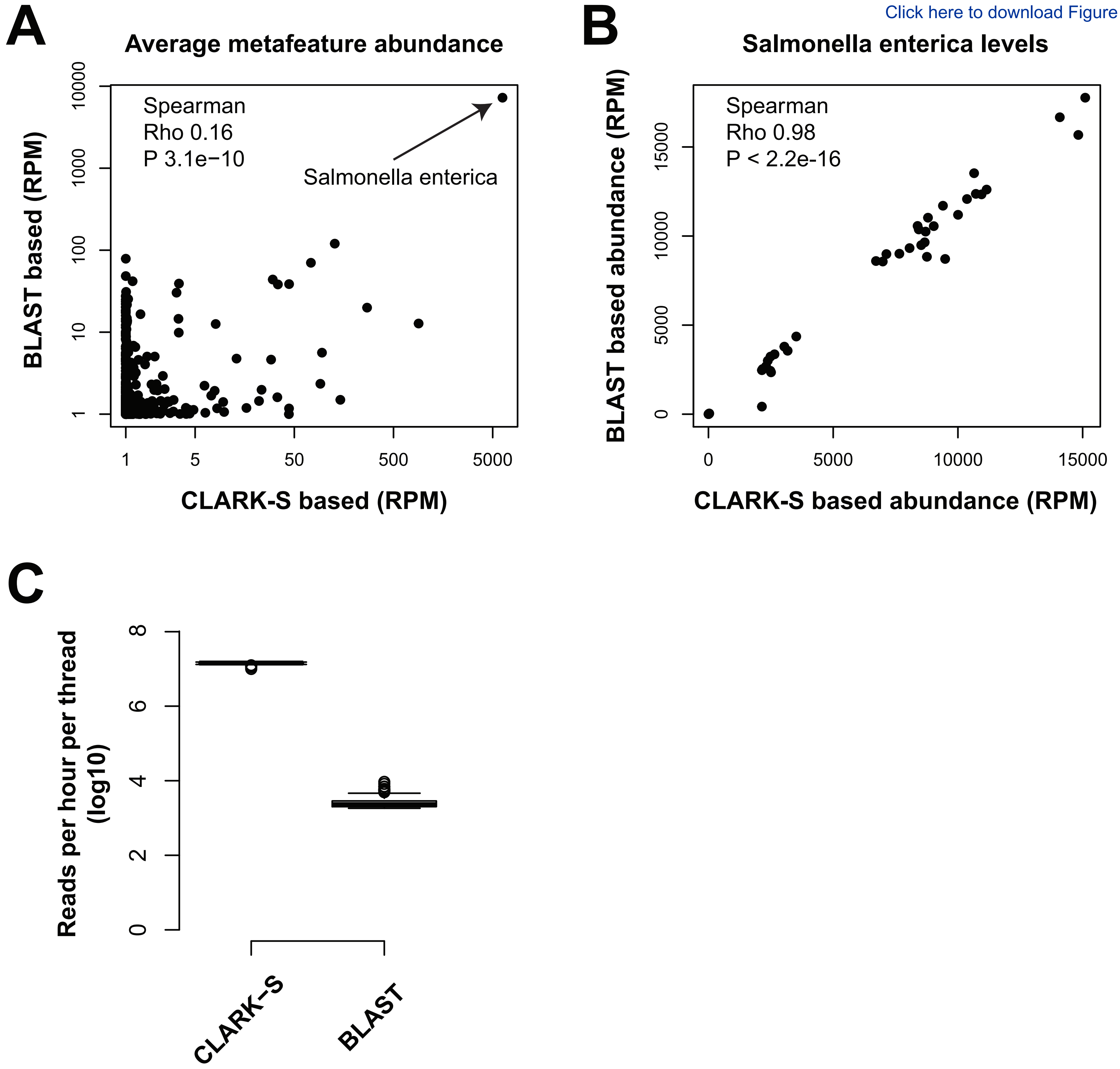

Additional file 1

Click here to access/download
Supplementary Material
AdditionalFile1.csv
