# Supplementary material for: MetaMap: an atlas of metatranscriptomic reads in human disease-related RNA-seq data
Source: Gigascience. 2018 Jun 12;7(6):giy070. doi: 10.1093/gigascience/giy070 (PMC6025204; doi:10.1093/gigascience/giy070)
Supplement: Response_to_Reviewer_Comments_Original_Submission.pdf [file giy070_response_to_reviewer_comments_original_submission.pdf]

Author's Response To Reviewer Comments

Close

Dear Editor,

We are resubmitting a revised and improved version of our manuscript. We would like to thank you and the reviewers for the valuable feedback. We have considered all points raised by the reviewers and we believe that we have addressed them all.
Our point by point answers are the following:

Reviewer reports:
Reviewer #1: This is an interesting idea, implementation and resource, which is useful and valuable. There are some possible confounders in this sort of analysis, they must be spelled out clearly, especially if the resource offers statistical analysis; it is not fully clear from the MS.

Reply: We appreciate the overall positive comment of the reviewer.

Changes needed:
- State clearly what is offered. Just the datasets of identified non-human reads? Or also the tool to generate equivalent data for new datasets? And further, also the pipeline for statistical analysis?
-- At least the second should be offered - scripts and/or tutorial for how to build DB-compatible similar output from a new RNA-seq dataset.

Reply: We have created a github repository (https://github.com/theislab/MetaMap) which contains a tutorial R script describing the statistical analysis and code to process new data. In total, the manuscript offers three outputs: 1) script to process new datasets with the MetaMap pipeline, 2) the count matrix derived from running the MetaMap pipeline on the described set of >17,000 samples and 3) example code for the statistical analysis.
To clarify this, we added the following sentence at the end of the Conclusions section of the abstract: “Additionally, codes to process new datasets and perform statistical analyses are made available at https://github.com/theislab/MetaMap.” In addition, we edited the last sentences of the manuscript as follows: “The composite metafeature OTU count table, derived from 17,278 cDNA libraries from 436 SRA projects, including annotations is provided for download [37]. The MetaMap pipeline and example code for performing statistical analysis can be found at https://github.com/theislab/MetaMap.”

- If the differential analysis pipeline is not offered to the reader, do not state there is any way to acquire significance for an enrichment of a non-human entity; better yet, provide those scripts.
-- If you do however, you must state precisely what significance is being tested, and how that is impacted by the potential issues listed below.

Reply: We acknowledge the concern of the reviewer regarding technical confounders. This is indeed an important point. As the reviewer pointed out in her last comment, within study case-control comparisons of each metafeature in turn are likely robust with regards to these concerns.
Therefore, we have added the following sentence to the Data validation and quality control

section:
“However, comparisons across studies and metafeatures may be biased by technical confounders (discussed in detail in the Re-use potential section). Therefore, we focussed our analysis on the comparison of a single metafeature across subjects within a study”
Additionally, we have added the following detailed discussion of the confounders and recommended analyses to the Re-use potential section:
“In addition, technical confounders can obstruct the analysis and potentially generate artificial differences if not considered properly. For example, different types of human samples may contain different amounts of non-human material due to varying sterility of the tissues. Furthermore, sequencing depth may introduce a detection floor for low abundant metafeatures. Therefore, comparisons across different tissues and sequencing depths may generate artificial differences. Additionally, given that only uniquely discriminative sequences are counted, the absolute abundance levels may not be comparable across metafeatures. Finally, the MetaMap pipeline captures metafeature abundance at the RNA level, which may not necessarily correspond to genomic abundance levels. Metafeatures may be low abundant at the DNA level but highly transcriptionally active and thus abundantly detected at the RNA level, or the inverse. These potential challenges need to be taken into consideration when comparing across metafeatures.
To minimize these effects, we encourage focusing on studies including intra-project comparisons testing each metafeature at a time, such as exemplified in the differential metafeature abundance analysis. Our rationale is that technical confounders - in contrast to biologically meaningful changes - should affect all runs within a project to the same extent and therefore not show condition-specific effects. For example, in the Westermann et al study [33] we detected substantial levels of phiX in both conditions (infected samples and mock-treated controls), but only the ‘Salmonella’ metafeature showed a condition-specific effect. We aim to address the challenges inherent to inter-project and inter-metafeature comparisons in future work.”

- There are potential confounders, and these must be discussed how they impact the sort of analyses you suggest, both generally and in your benchmark.
-- First, different types of human samples will have different amounts of non-human materials because the compartments are more or less sterile, regardless of differential clinical risk etc. This may also reflect purely technical considerations such as risk of contamination for different types of sampling etc. How is this accounted for, and what possible impact may it have?

Reply: The reviewer makes a valid point. Varying compartments can lead to varying sterility. However, we make the assumption that the studies were designed in such a way that technical factors, such as cellular compartment, are not confounded with the conditions being tested. Therefore, we encourage comparisons across samples obtained from the same study which are likely to be robust to these technical confounders. To make this clear we have added detailed discussion to the re-use potential section (see details above).

-- One particularly important effect is that low abundance means a detection floor, where some samples have more things getting above that floor, so one would have a risk of artificial signal resulting just from sample size or technical factors. There would be ways around that but it must be considered and discussed.

Reply: The reviewer makes a valid point. Low abundance can lead to a detection floor. However, we make the assumption that the studies were designed in such a way that

technical factors, sequencing depth, are not confounded with the conditions being tested. Therefore, we encourage comparisons across samples obtained from the same study which are likely to be robust to these technical confounders. To make this clear we have added detailed discussion to the re-use potential section (see details above).

-- For RNA being expression data, gene expression (as a transient, condition-dependent thing) will have additional impact beyond the abundance of non-human entitities genomically, however you assess taxonomic composition/presence. This may introduce biases (detected features may be low-abundant but highly active, or the inverse), and different genes in those entities may show up more or less abundantly. This too must be discussed and considered.

Reply: We agree with the reviewer that the manuscript needs to clarify the difference between measuring DNA and RNA abundance. To make this clear we have added detailed discussion to the re-use potential section (see details above).

-- How database-sensitive is the CLARK-S tool? I.e. will many more non-human entities be found if they come from well-studied, well-represented clades? The risk seems larger still if it aims for species-specific marker sequences; it seems then likely to miss entirely sisters or cousins of the already-known features, which would cause artificial difference/contrast.

Reply: The reviewer makes a good argument. Absolute comparisons across metafeatures can be biased. Therefore, we encourage comparisons of the same metafeature across samples which are likely to be robust to this technical confounder. To make this clear we have added detailed discussion to the re-use potential section (see details above).

-- How does one normalize these sorts of data for inter-sample comparisons?

Reply: The data are "normalized" during statistical comparison by including covariates (such as sequencing depth) into the model. We use the DESeq2 R package to perform the statistical comparison and by default DESeq2 normalizes for library size differences by including the estimated size factors as covariates in the model. To clarify this we edited the following sentence in the Data validation and quality control section:
"Using the annotation provided in the respective study, we performed differential metafeature abundance analysis to identify those metafeatures that show the largest relative difference in abundance levels between the infected and control samples (see Methods for details)."
Moreover, we added the following sentences to the Differential metafeature abundance section: "DESeq2 models differential gene expression by fitting a negative binomial distribution to the raw counts underlying RNA-seq data. This framework can account for confounding variables such as sequencing depth. Therefore, the data need not be normalized prior to statistical inter-sample comparisons."

-- For analysing equivalent sample types (e.g. treated/untreated, testing each feature in turn) this is less of a problem, whereas if one e.g. compares viral load in different tissues, or compare prevalence of different viruses, it becomes more of a problem. These are not challenges the authors must solve, necessarily, but to make the tool maximally useful (including as a starting point for future work) these challenges must be discussed and acknowledged in detail.

Reply: The reviewer has pointed out a number of important challenges that need to be considered during the analysis of this data. We have extended the re-use potential section to discuss the technical confounders and suggest analysis approaches to make the tool maximally useful (see details above).

Reviewer #2: The authors have developed a valuable database of viral, bacterial and fungal sequences derived from publically available human RNA-seq datasets. They have carefully selected RNA-seq datasets that are not poly-A selected (to avoid datasets that will be primary human based on molecular enrichment). The methodology they present is sound, and the experiments that they designed to validate the data are convincing.

As noted by the editor the database is not currently available, but once this is amended, the publication looks to be ready. I would also recommend that the protocol in protocols.io is also update with this important information upon publication.

Reply: We thank the reviewer for the positive review. We have deposited the data set in the GigaScience Database and added the following Availability of supporting data section to the manuscript:
"The data sets supporting the results of this article are available in the GigaScience Database repository, http://dx.doi.org/10.5524/100456."

Close
