# Supplementary material for: MetaMap: an atlas of metatranscriptomic reads in human disease-related RNA-seq data
Source: Gigascience. 2018 Jun 12;7(6):giy070. doi: 10.1093/gigascience/giy070 (PMC6025204; doi:10.1093/gigascience/giy070)
Supplement: Reviewer_1_Report_(Original_Submission) -- Kristoffer Forslund — 3/5/2018 Reviewed [file giy070_reviewer_1_report_(original_submission).pdf]

Reviewer Report

Title: **MetaMap: An atlas of metatranscriptomic reads in human disease-related RNA-seq data"**

Version: **Original Submission** **Date:** 3/5/2018

Reviewer name: **Kristoffer Forslund**

Reviewer Comments to Author:

This is an interesting idea, implementation and resource, which is useful and valuable. There are some possible confounders in this sort of analysis, they must be spelled out clearly, especially if the resource offers statistical analysis; it is not fully clear from the MS.Changes needed:- State clearly what is offered. Just the datasets of identified non-human reads? Or also the tool to generate equivalent data for new datasets? And further, also the pipeline for statistical analysis?-- At least the second should be offered - scripts and/or tutorial for how to build DB-compatible similar output from a new RNA-seq dataset.- If the differential analysis pipeline is not offered to the reader, do not state there is any way to acquire significance for an enrichment of a non-human entity; better yet, provide those scripts.-- If you do however, you must state precisely what significance is being tested, and how that is impacted by the potential issues listed below.- There are potential confounders, and these must be discussed how they impact the sort of analyses you suggest, both generally and in your benchmark.-- First, different types of human samples will have different amounts of non-human materials because the compartments are more or less sterile, regardless of differential clinical risk etc. This may also reflect purely technical considerations such as risk of contamination for different types of sampling etc. How is this accounted for, and what possible impact may it have?-- One particularly important effect is that low abundance means a detection floor, where some samples have more things getting above that floor, so one would have a risk of artificial signal resulting just from sample size or technical factors. There would be ways around that but it must be considered and discussed.-- For RNA being expression data, gene expression (as a transient, condition-dependent thing) will have additional impact beyond the abundance of non-human entitities genomically, however you assess taxonomic composition/presence. This may introduce biases (detected features may be low-abundant but highly active, or the inverse), and different genes in those entities may show up more or less abundantly. This too must be discussed and considered.-- How database-sensitive is the CLARK-S tool? I.e. will many more non-human entities be found if they come from well-studied, well-represented clades? The risk seems larger still if it aims for species-specific marker sequences; it seems then likely to miss entirely sisters or cousins of the already-known features, which would cause artificial difference/contrast.-- How does one normalize these sorts of data for inter-sample comparisons?-- For analysing equivalent sample types (e.g. treated/untreated, testing each feature in turn) this is less of a problem, whereas if one e.g. compares viral load in different tissues, or compare prevalence of different viruses, it becomes more of a problem. These are not challenges the authors must solve, necessarily, but to make the tool maximally useful (including as a starting point for future work) these challenges must be discussed and acknowledged in detail.

Level of Interest

Please indicate how interesting you found the manuscript: An article of importance in its field

Quality of Written English

Please indicate the quality of language in the manuscript: Acceptable

Declaration of Competing Interests

Please complete a declaration of competing interests, considering the following questions:

- Have you in the past five years received reimbursements, fees, funding, or salary from an organisation that may in any way gain or lose financially from the publication of this manuscript, either now or in the future?
- Do you hold any stocks or shares in an organisation that may in any way gain or lose financially from the publication of this manuscript, either now or in the future?
- Do you hold or are you currently applying for any patents relating to the content of the manuscript?
- Have you received reimbursements, fees, funding, or salary from an organization that holds or has applied for patents relating to the content of the manuscript?
- Do you have any other financial competing interests?
- Do you have any non-financial competing interests in relation to this paper?

If you can answer no to all of the above, write 'I declare that I have no competing interests' below. If your reply is yes to any, please give details below.

I declare that I have no competing interests.

I agree to the open peer review policy of the journal. I understand that my name will be included on my report to the authors and, if the manuscript is accepted for publication, my named report including any attachments I upload will be posted on the website along with the authors' responses. I agree for my report to be made available under an Open Access Creative Commons CC-BY license (http://creativecommons.org/licenses/by/4.0/). I understand that any comments which I do not wish to be included in my named report can be included as confidential comments to the editors, which will not be published.

I agree to the open peer review policy of the journal
