# Supplementary material for: MetaMap: an atlas of metatranscriptomic reads in human disease-related RNA-seq data
Source: Gigascience. 2018 Jun 12;7(6):giy070. doi: 10.1093/gigascience/giy070 (PMC6025204; doi:10.1093/gigascience/giy070)
Supplement: Reviewer_2_Report_(Original_Submission) -- Bonnie Hurwitz — 4/19/2018 Reviewed [file giy070_reviewer_2_report_(original_submission).pdf]

Reviewer Report

Title: MetaMap: An atlas of metatranscriptomic reads in human disease-related RNA-seq data

Version: Original Submission **Date:** 4/19/2018

Reviewer name: Bonnie Hurwitz

Reviewer Comments to Author:

The authors have developed a valuable database of viral, bacterial and fungal sequences derived from publically available human RNA-seq datasets. They have carefully selected RNA-seq datasets that are not poly-A selected (to avoid datasets that will be primary human based on molecular enrichment). The methodology they present is sound, and the experiments that they designed to validate the data are convincing. As noted by the editor the database is not currently available, but once this is amended, the publication looks to be ready. I would also recommend that the protocol in protocols.io is also update with this important information upon publication.A valuable resource!

Level of Interest

Please indicate how interesting you found the manuscript: Choose an item.

Quality of Written English

Please indicate the quality of language in the manuscript: Choose an item.

Declaration of Competing Interests

Please complete a declaration of competing interests, considering the following questions:

- Have you in the past five years received reimbursements, fees, funding, or salary from an organisation that may in any way gain or lose financially from the publication of this manuscript, either now or in the future?
- Do you hold any stocks or shares in an organisation that may in any way gain or lose financially from the publication of this manuscript, either now or in the future?
- Do you hold or are you currently applying for any patents relating to the content of the manuscript?
- Have you received reimbursements, fees, funding, or salary from an organization that holds or has applied for patents relating to the content of the manuscript?
- Do you have any other financial competing interests?
- Do you have any non-financial competing interests in relation to this paper?

If you can answer no to all of the above, write 'I declare that I have no competing interests' below. If your reply is yes to any, please give details below.

I declare that I have no competing interests

I agree to the open peer review policy of the journal. I understand that my name will be included on my report to the authors and, if the manuscript is accepted for publication, my named report including any attachments I upload will be posted on the website along with the authors' responses. I agree for my report to be made available under an Open Access Creative Commons CC-BY license (http://creativecommons.org/licenses/by/4.0/). I understand that any comments which I do not wish to be included in my named report can be included as confidential comments to the editors, which will not be published.

I agree to the open peer review policy of the journal
